# Transforming epitope-specific gp120 monomer-based probes into immunogens with N-linked glycan masking

**DOI:** 10.1186/1742-4690-9-S2-P318

**Published:** 2012-09-13

**Authors:** G Chuang, JC Boyington, GJ Nabel, PD Kwong, I Georgiev

**Affiliations:** 1 National Institutes of Health/NIAID, Bethesda, MD, USA

## Background

HIV-1 gp120 monomer-based probes have been used for the identification of broadly neutralizing antibodies. Such probes could represent starting points in the design of HIV-1 immunogens, though efforts must be made to silence immune responses directed toward non-neutralizing epitopes. One possible approach would be to mask these epitopes by introducing N-linked glycan. A potential complication to such an approach is glycan occupancy: although N-linked glycosylation generally occurs at N-X-T/S sequons, many such sequons are not occupied.

## Methods

A computational protocol was developed to identify the putative positions for insertion of N-linked glycan on the gp120 surface. The first step involves the identification of residue positions on the gp120 surface where the insertion of the N-X-T/S sequon is predicted as energetically-tolerable. The second step involves the application of NGlycPred, a Random Forest-based predictor, to predict the glycan occupancy at the inserted sequons.

## Results

The glycan occupancy prediction of the protocol is highly correlated to validated N-X-T sequon insertion designs. Multiple sequon insertions to gp120 monomer-based probes were generated based on the protocol.

## Conclusion

A computational protocol was implemented to identify putative sites for insertion of N-X-T/S sequons with improved likelihood of glycan occupancy. The protocol is applicable to the design of N-linked glycans for masking non-neutralizing antibody epitopes on gp120-based probes as well as other immunogen candidates.

